# The impact of perceived volume limits on subcutaneous biologic drug development: an industry survey

**DOI:** 10.1080/10717544.2026.2756024

**Published:** 2026-11-07

**Authors:** Mehul Desai, Wei Chen, Maxwell Koobatian, Deepa Rajagopalan, Shirish Ingawale, Deep Bhattacharya

**Affiliations:** a Enable Injections, Inc., Cincinnati, Ohio, USA; b CMC and Drug Device Combination Products, WuXi Biologics USA, San Francisco, California; c Corporate Development, Crinetics Pharmaceuticals, San Diego, California; d Enveda Biosciences, Boulder, Colorado, USA; e Takeda Pharmaceuticals, Boston, Massachusetts, USA; f Pfizer, Inc, Andover, Massachusetts, USA

**Keywords:** Biologic drugs, subcutaneous administration, large-volume, drug development, drug formulation, survey

## Abstract

As multiple large-volume subcutaneous (SC) biologics enter trials or gain approval, understanding how administration volume impacts biologic development is critical. While patients often prefer SC over intravenous administration, large-volume SC delivery adoption is hindered by the belief that SC delivery must be ≤3 mL without a permeation enhancer (PE). A 21-item online survey was conducted among professionals working in research and development; chemistry, manufacturing, and controls (CMC); portfolio strategy, new product planning; and combination products. Questions explored perceptions of SC administration volume limits, biologic deprioritization and abandonment, large-volume delivery considerations, and organizational approaches to revisiting deprioritized candidates. Eighty-eight participants completed the survey; 90.9% reported direct involvement in at least one project in which a biologic had been deprioritized or abandoned due to challenges achieving an SC volume of ≤3 mL. Respondents estimated that a weighted mean of 32.4% of relevant biologics had been abandoned and 28.9% had been deprioritized because of such challenges. Frequently selected reasons included limited experience with large-volume formulations or devices (29.5%), patient and/or healthcare provider preference (19.3%), and development costs or timelines (17.0%). Overall, 33.0% of respondents selected ≤3 mL as the maximum volume for SC infusion over several minutes without a PE. These findings indicate that perceived SC volume constraints may influence development decisions and highlight the importance of differentiating between device-related and physiological limits on SC administration volume. This study demonstrates that misconceptions about SC injection volume limits may lead to deprioritization or abandonment of viable assets during biologic development.

## 1. Introduction

Subcutaneous (SC) administration of biologic drugs is an area of growing clinical and regulatory focus, with treatment options expanding rapidly across multiple therapeutic areas, including oncology and autoimmune diseases (Martins et al. 2022). These biologics are typically administered intravenously (IV) by healthcare providers (HCPs), but SC administration has several advantages over IV administration, including faster administration, improved tolerability, decreased dosing errors, patient preference, and the facilitation of self-administration (Pivot et al. 2014; Overton et al. 2021; Usmani et al. 2022; Desai et al. 2023).

One key challenge in formulating SC biologics is their reduced bioavailability compared with IV administration (Richter et al. 2012). To compensate, higher total doses are often required for SC delivery, leading manufacturers to increase formulation concentration to minimize injection volume. However, increasing concentration can introduce formulation challenges, and required administration volumes may exceed the capacity of some conventional SC delivery devices. Several large-volume SC biologics have received regulatory approval, including ravulizumab (7 mL), (Kulasekararaj et al. 2025) pegcetacoplan (20 mL), (Hoy 2021) and immune globulin subcutaneous (human), 20% liquid (≤25 mL/site for primary immunodeficiency or ≤50 mL/site for chronic inflammatory demyelinating polyneuropathy) and several others. (U.S. Food and Drug Administration 2010) Additionally, isatuximab is available as a 10-mL SC formulation for the treatment of multiple myeloma delivered via an on-body injector (OBI, also called on-body delivery systems or wearable injectors) (Sanofi 2026; U.S. Food and Drug Administration, 2026). Multiple large-volume SC formulations are also in development without the use of permeation enhancers (PEs) (Green et al. 2024).

Collectively, these examples demonstrate that ≤3 mL is not a universal upper limit for SC administration. Feasible volume depends on anatomical site, injection rate and duration, device design, formulation concentration and viscosity, and patient characteristics, while systemic exposure depends on the administered dose and its bioavailability. These approvals, dating back almost two decades, demonstrate that very large volumes (e.g. 50 mL per SC injection site) have long been feasible without use of PE such as recombinant human hyaluronidase (e.g. rHuPH20 or ALT-B4). The absence of a universal SC volume limit is also supported outside the biologics field; a recent systematic review of subcutaneously administered beta-lactam antibiotics concluded that SC administration is a viable route with generally mild adverse events and acceptable pharmacokinetic profiles (Moreal et al. 2024).

Practical SC volume constraints differ according to the method and duration of administration. Bolus administration over seconds and SC infusion over several minutes should therefore not be assumed to have equivalent volume constraints. Available delivery approaches include manual syringes, autoinjectors, infusion pumps, and OBIs, which differ in device capacity, administration rate and duration, and user requirements. Formulation-based strategies, including increased biologic concentration or use of PEs, may also alter the volume or conditions required for SC administration. Thus, a volume threshold relevant to one administration approach should not necessarily be interpreted as a universal physiological limit; such constraints are attributes of the delivery technology rather than of subcutaneous tissue itself.

While 2-3 mL is the current practical limit for SC bolus injections, and conventional SC autoinjectors typically have volume capacities of 1-2.25 mL, newer drug delivery devices have substantially larger volume capacity. A comparison between weekly autoinjectors and four-weekly OBIs can be found in Figure 1. OBIs can deliver 0.6-25 mL and allow for administration of low-concentration, large-volume drug formulations; autoinjectors are well-established, can be administered by individuals with no formal medical training, and are reported to be easy to use (Dostal et al. 2023). Although disposable handheld autoinjectors are moving toward a 5.5 mL capacity, they still require manual administration, and there is a practical ceiling on how long a patient can be expected to hold a device steady against their skin (Figure 2) (Parker et al. 2023; Boyd 2023). Like SC pumps, OBIs offer hands-free, large-volume administration; they also avoid the need to include a permeation enhancer in formulations while remaining compatible with PE-coformulated drugs when needed. One way to potentially improve patient comfort is to use adaptive drug delivery systems that modulate flow rate in response to real-time SC backpressure, rather than fixed spring-driven mechanisms that deliver with substantial force (Figure 2) (Doughty et al. 2016; Kim et al. 2017; Pepin et al. 2023). Nevertheless, misconceptions regarding SC volume limits persist and may influence drug discovery and development strategies (Figure 3) (Desai et al. 2025b, 2026).

**Figure 1.**
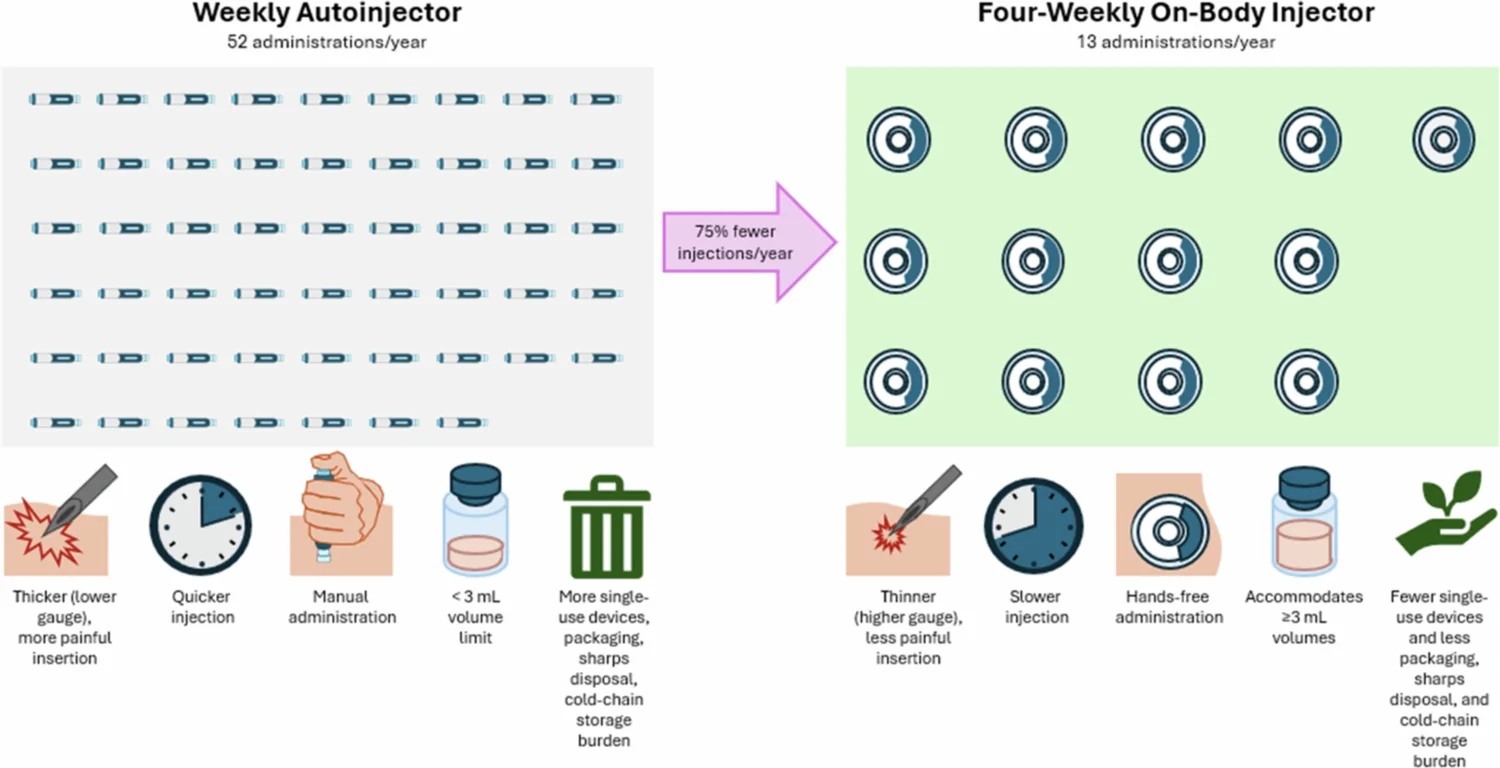
Benefits and drawbacks of weekly autoinjectors vs. four-weekly on-body injectors. Abbreviations: mL, milliliters.

**Figure 2.**
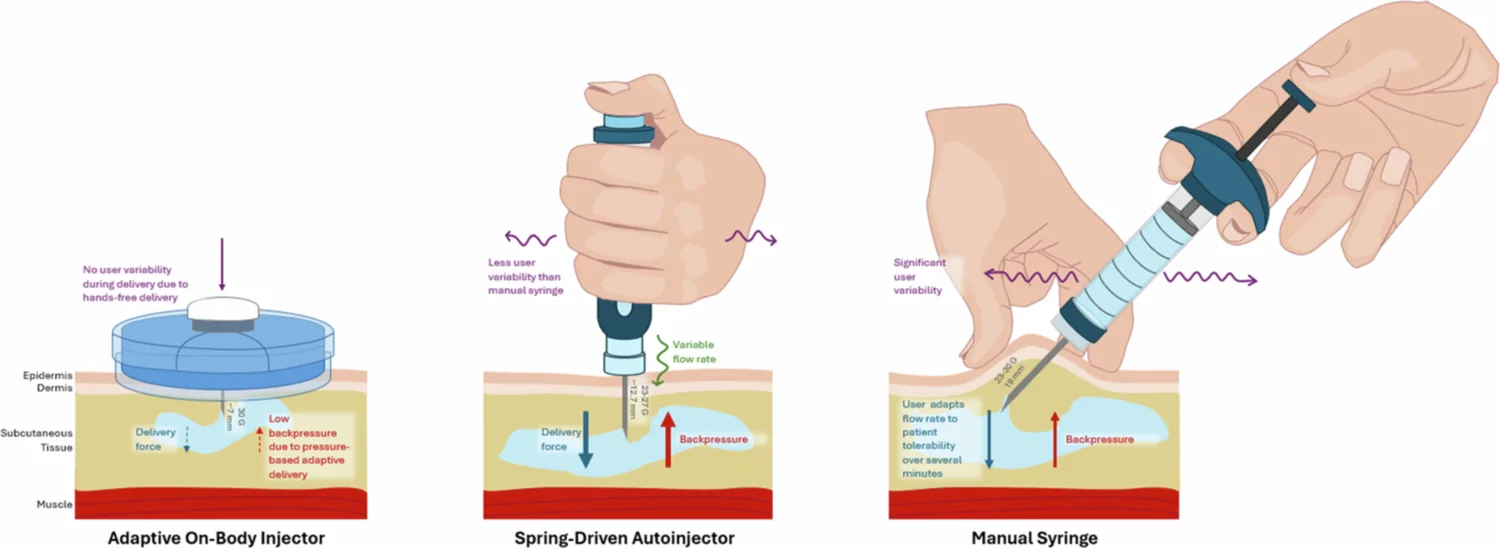
Comparison among adaptive on-body injector, spring-driven autoinjector, and manual syringe with respect to needle gauge and length, user variability, delivery force, flow rate, and backpressure. Abbreviations: G, gauge; mm, millimeters.

**Figure 3.**
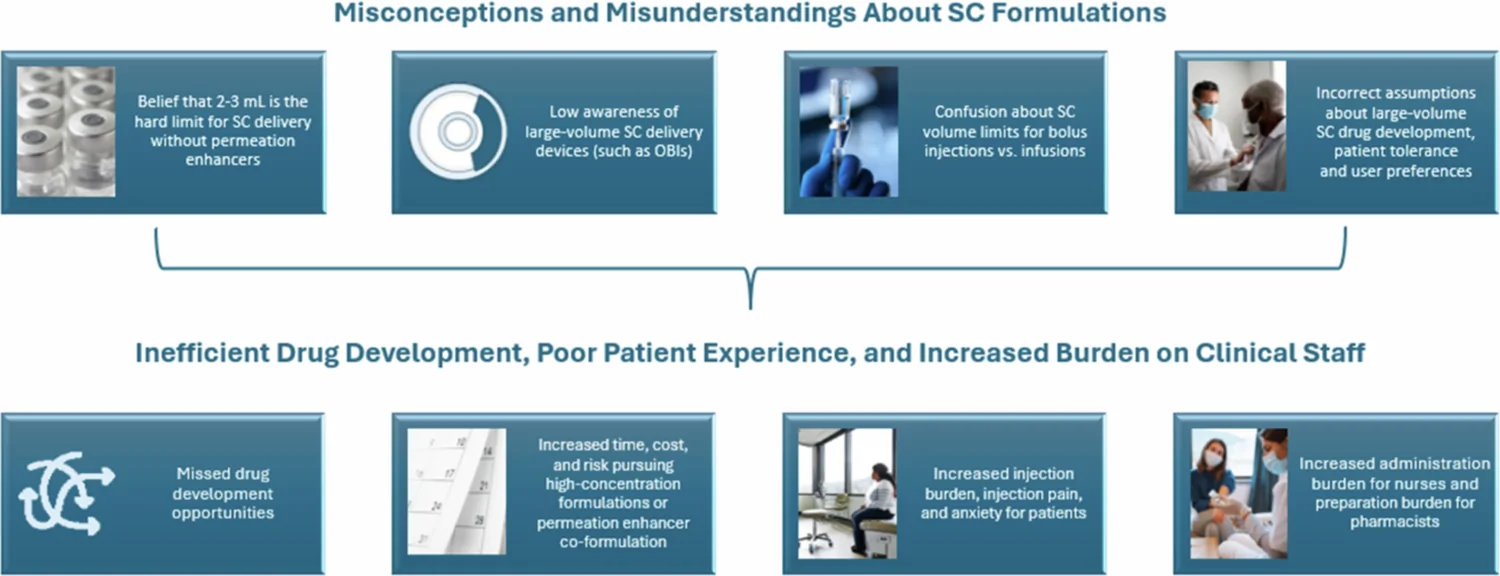
Impact of misconceptions and misunderstandings about SC formulations. Abbreviations: mL, milliliters; OBI, on-body injector; SC, subcutaneous.

High-concentration SC biologic development can present technical challenges and is associated with meaningful development delays. In a previous survey of drug formulation experts, 69% of respondents reported delays in clinical trials or product launches attributable to challenges in developing high-concentration SC formulations (Desai et al. 2025b). Among respondents reporting delays, the weighted mean delay was 11.3 months, demonstrating the impact that high-concentration formulation challenges can have on development timelines (Desai et al. 2025b). The most frequently cited challenges included aggregation, viscosity, and solubility limitations, all of which are exacerbated by increased drug concentration (Desai et al. 2025b). These issues reflect the interdependence of formulation variables such as bioavailability, osmolality, tonicity, volume, and pH, (Desai et al. 2023; Davis et al. 2024) and help explain why an exclusive focus on achieving ≤ 3 mL SC injection volumes through concentration can be costly, time-consuming, and high-risk (Mathaes et al. 2016; Desai et al. 2023).

Drug development programs may be deprioritized or discontinued for numerous scientific, technical, strategic, and commercial reasons. Among the technical reasons occasionally disclosed are formulation barriers, as with a monoclonal antibody candidate discontinued because of low solubility, increased aggregation, and high viscosity (Nichols et al. 2015). Some subsequently undergo further development following licensing, acquisition, repurposing, or changes in development strategy (Table 1). These examples are not intended to attribute the original development decisions to SC administration volume constraints, which are generally not publicly reported, but rather to illustrate that deprioritized assets may subsequently re-enter development. However, the extent to which perceived SC administration volume constraints contribute to such decisions has not been well characterized. Biopharmaceutical companies frequently shelve biologic candidates not because of insufficient efficacy or safety but due to strategic deprioritization, formulation limitations, or market dynamics.

**Table 1.** Examples of deprioritized or discontinued drug development programs subsequently advanced by other organizations.

Drug	Earlier development/program status	Subsequent developer	Development outcome
Baxdrostat (aldosterone synthase inhibitor)	Roche licensed the compound to CinCor Pharma in 2019 (CinCor Pharma 2019). A Phase 2 study in treatment-resistant hypertension subsequently missed its primary endpoint, although a subgroup showed benefit (Freeman et al. 2023).	CinCor Pharma; subsequently AstraZeneca	AstraZeneca acquired CinCor in 2023 and continued development of baxdrostat in cardiorenal disease (Taylor 2023).
Bimagrumab (anti-activin type II receptor antibody)	Novartis discontinued development following studies in muscle-wasting disorders (Hanna et al. 2019).	Versanis Bio; subsequently Eli Lilly	Versanis advanced bimagrumab for obesity; Eli Lily acquired Versanis Bio in 2023 and continued the program (Eli Lilly and Company 2023; Eli Lilly and Company 2023).
Lebrikizumab (anti-IL-13 antibody)	Roche discontinued/divested the program after mixed Phase 3 results in asthma (Gray 2016).	Dermira; subsequently Eli Lilly	Dermira advanced lebrikizumab in atopic dermatitis after licensing worldwide rights in 2017 (Dermira 2017). Following Lilly’s acquisition of Dermira, lebrikizumab received FDA approval in 2024 (Eli Lilly and Company 2020; Center for Drug Evaluation and Research (CDER) 2024; FDA 2024).
Mirdametinib (small molecule MEK inhibitor)	SpringWorks obtained mirdametinib from Pfizer in 2017.	SpringWorks Therapeutics; subsequently Merck KGaA	SpringWorks advanced the compound in neurofibromatosis type 1, leading to FDA approval in 2025 (SpringWorks 2025; U.S. Food and Drug Administration 2025; Armstrong 2025). Merck KGaA subsequently acquired SpringWorks (Merck KGaA 2025).
Momelotinib (JAK1/2 and ACVR1 inhibitor)	Following development of Cytopia/YM Biosciences and subsequently Gilead, Sierra Oncology acquired momelotinib from Gilead in 2018 (YM BioSciences 2010; Sierra Oncology 2018).	Sierra Oncology; subsequently GlaxoSmithKline	Momelotinib was advanced through Phase 3 development in myelofibrosis and received FDA approval in 2023 (Ojjaara (momelotinib) 2023).
Obicetrapib (CETP inhibitor)	Amgen discontinued development of obicetrapib in 2017 (Forbion 2020).	NewAmsterdam Pharma	NewAmsterdam Pharma acquired the compound in 2020 (Forbion 2020) and advanced it into Phase 3 development for low-density lipoprotein cholesterol reduction and MACE risk reduction; (European Medicines Agency for Obicetrapib 2025; Sarraju et al. 2025) European marketing applications were accepted for review in 2025 (European Medicines Agency for Obicetrapib 2025).
Tapinarof (aryl hydrocarbon receptor agonist)	Following earlier development by GlaxoSmithKline, Dermavant acquired tapinarof in 2018.	Dermavant Sciences; subsequently Organon	Dermavant advanced tapinarof through Phase 3 development in plaque psoriasis, (Lebwohl et al. 2021) leading to FDA approval in 2022. (Vinluan 2022) Organon subsequently acquired Dermavant and tapinarof (Kansteiner 2024).
Teplizumab (anti-CD3 antibody)	After a Phase 3 study conducted with Eli Lilly failed its primary endpoint (MacroGenics 2010), Provention Bio acquired rights to teplizumab from MacroGenics in 2018.	Provention Bio; subsequently Sanofi	Provention Bio advanced teplizumab in type 1 diabetes, achieving FDA approval in 2022; Sanofi subsequently acquired Provention Bio (Manalac 2022; Sanofi 2023).
Vibegron (β3-agonist)	Merck developed vibegron through Phase 3 in overactive bladder before discontinuing the program as it exited urology (Adams 2017).	Urovant Sciences; subsequently Sumitovant (Sumitomo)	Urovant Sciences licensed vibegron from Merck in 2017, (Adams 2017) conducted additional Phase 3 development, (Staskin et al. 2020) and achieved FDA approval in December 2020 (Sumitovant BioPharma 2021).
Xanomeline (muscarinic agonist)	Development of xanomeline was limited by gastrointestinal adverse events despite evidence of antipsychotic activity (Bodick et al. 1997).	Karuna Therapeutics; subsequently Bristol Myers Squib	Karuna Therapeutics combined xanomeline with trospium to mitigate adverse effects. The combination demonstrated efficacy in schizophrenia (Correll et al. 2022; Kishi et al. 2025; Żełabowski et al. 2026) and subsequently received regulatory approval (Syed 2025).

Note: These examples illustrate that deprioritized or discontinued biologic development programs may subsequently re-enter development following licensing, acquisition, repurposing, or changes in development strategy. Abbreviations: ACVR, activin A receptor type 1; CD3, cluster of differentiation 3; CETP, cholesteryl ester transfer protein; FDA, Food and Drug Administration; IL, interleukin; JAK, Janus kinase proteins; MACE, major adverse cardiovascular events; MEK, mitogen-activated extracellular kinase; NSCLC, non-small-cell lung cancer; PARP, poly (adenosine diphosphateribose) polymerase.

Given gaps in the literature regarding the influence of administration volume on SC biologic development decisions, this study investigated industry professionals’ perceptions of feasible SC administration volumes and their reported experiences of how volume-related challenges may impact drug prioritization and development.

## 2. Materials and methods

### 2.1. Study design

This descriptive, cross-sectional study enrolled a sample of respondents who met prespecified eligibility criteria designed to identify professionals with experience in SC biologic drug development and related decision-making. The survey was conducted in November 2024 and was administered by a third-party vendor engaged and funded by Enable Injections, Inc. The survey instrument was designed by the authors; the vendor’s role was limited to survey administration and provision of the resulting survey response data. Respondents were not informed of the identity of the study sponsor during survey participation.

### 2.2. Participants

The target population comprised experienced biotechnology and pharmaceutical industry professionals working in functions relevant to SC biologic development, including research and development (R&D); chemistry, manufacturing, and controls (CMC); portfolio strategy/new product planning; and combination products.

The survey vendor maintains a panel of >1.5 million registered respondents. The survey invitation was distributed to the vendor’s full panel, and respondents who elected to participate completed a series of screening questions to determine eligibility.

Excluded participants included those who indicated specialization in medical affairs or commercial functions; those with two or fewer years of experience in the field or experience working on four or fewer high-concentration SC drug products; those self-reporting minimal or low familiarity with the development of high-concentration (≥100 mg/mL) SC formulations or understanding of the challenges associated with their development; those self-reporting no or minimal familiarity with the development and outcomes of drugs requiring large volumes for SC injection; and those reporting no involvement with, or uncertainty about involvement with, projects where drugs were deprioritized or abandoned due to challenges in achieving small (≤3 mL) volumes for SC administration or with decision-making regarding SC drug product development. Responses deemed invalid by the vendor, such as invariant responses across questions or nonsensical responses to free-text items, were also excluded.

Because participation depended on panel members electing to enter the survey and subsequently meeting the restrictive eligibility criteria, the final sample represents a self-selected group of eligible industry professionals and was not intended to be representative of the vendor panel or the wider biopharmaceutical industry.

### 2.3. Survey

The questionnaire comprised 14 screening and demographic questions and 21 survey questions (Supplementary Material). Questions explored perceptions of SC administration volume thresholds, respondent-reported experiences of drug candidate deprioritization or abandonment, considerations related to large-volume delivery, and organizational approaches to tracking and revisiting deprioritized candidates. Question types included multiple-choice items, Likert scales (where, e.g. 1 = Not at all familiar and 5 = Extremely familiar), percentage range response options, and free-text answers.

For the purposes of the survey, large-volume SC administration was operationally defined as ≥ 3 mL. This threshold was used to define the volume range examined in the questionnaire and should not be interpreted as a universal physiological or device-independent upper limit for SC administration. The survey separately assessed perceived volume limits for SC bolus administration and SC infusion over several minutes. Accordingly, references to SC infusion in the reporting of survey findings refer specifically to infusion over several minutes unless otherwise stated. The investigator-developed questionnaire underwent pilot testing and internal expert review but did not undergo cognitive testing or formal validation before administration.

### 2.4. Ethics

This study involved an anonymous, voluntary, online survey of professionals working in the biopharmaceutical industry. No patients, healthcare providers in a clinical capacity, or vulnerable populations were surveyed. No personally identifiable information was collected at any point during the study, and all responses were recorded anonymously. This research is exempt from institutional review board oversight in accordance with the U.S. Code of Federal Regulations, Title 45, Part 46, Section 46.104(d)(2), which provides exemption for research involving survey procedures where (i) information is recorded in such a manner that the identity of subjects cannot readily be ascertained, and (ii) disclosure of responses outside the research would not reasonably place subjects at risk.

Before beginning the survey, participants were required to agree to the survey vendor’s standard compliance statement. This compliance statement included stipulations that participants would be compensated for their time, that they were legally allowed to disclose information asked about in the survey, and that they had read the vendor’s privacy policy. Respondents who chose not to agree to the compliance statement were unable to complete the survey and were excluded from the sample. A separate study-specific electronic consent statement required participants to confirm that by agreeing to participate, they had read and understood the purpose and details of this survey, voluntarily agreed to participate, and understood that their responses may be used for scientific and academic purposes. Participants who did not agree with this additional electronic consent statement were also excluded from the sample.

### 2.5. Statistical analysis

Complete survey responses were tabulated and summarized using descriptive statistics. No formal hypothesis testing or a priori sample size calculation was performed. Results are presented as number and percentage of participants (*n* [%]) or as weighted mean, median, and mode, as appropriate.

For questions with percentage ranges (e.g. 1−10%, 11−20%) rather than exact numerical response options, bootstrapping was used to estimate the median and its 95% confidence interval (CI). Each response range was defined by its lower and upper bounds and the number of respondents selecting that range. For each of 10,000 bootstrap replicates, response ranges were sampled in proportion to their observed frequencies, and values were sampled uniformly within the selected ranges. The median was calculated for each simulated sample. The mean of the resulting 10,000 medians was used as the point estimate, with 95% CIs derived from the 2.5^th^ and 97.5^th^ percentiles of the bootstrap distribution. This approach accounts for uncertainty arising both from sampling variation and interval-based responses without assuming a specific underlying distribution. Bootstrapping was performed using Python 3.13.5 within Spyder 6.0.7 with NumPy 2.1.3 and matplotlib 3.10.0.

## 3. Results

### 3.1. Participant demographics and characteristics

A total of 2241 individuals were excluded from the study due to their responses to screening questions; 88 participants completed the survey. Participant demographics and characteristics are presented in Table 2. Participants were located in the United States (52.3%), Europe (38.6%), or Asia (9.1%). Most (76.1%) had worked on 1–10 high-concentration SC biologicproducts during their career, with 15.9% having worked on 11–20, 3.4% each on 21–30 or 31−100, and 1.1% on > 100. About half (46.6%) were senior/mid-level management, 27.3% were technical experts or specialists involved in strategic decision-making, and 26.1% were executive/senior leadership. The size of biotech/pharma companies varied; 30.7% worked for mid-cap, 27.3% for large-cap, 19.3% for small-cap, and 5.7% for micro-cap. The remaining 17.0% worked for other organizations, such as contract research organizations, or were independent chemistry, manufacturing, and controls (CMC) consultants. Half of the participants worked in early research and development (R&D) (23.9%) or CMC (26.1%), with 18.2% working in pipeline/portfolio strategy, 17.0% in new product planning and 14.8% in combination products. Approximately half of respondents reported oncology as their main therapeutic area of experience with SC drug products (*n* = 46, 52.3%); most respondents reported rare disease (78.4%) or ultra-rare disease (2.3%) expertise.

**Table 2.** Participant demographics and characteristics.

Demographics and characteristics, *n* (%)	Participants (*N* = 88)
Geographic location	
United States	46 (52.3)
Europe	34 (38.6)
Asia	8 (9.1)
Organization	
Large-cap pharma/biotech (≥$10 billion market capitalization)	24 (27.3)
Mid-cap pharma/biotech ($2–$10 billion market capitalization)	27 (30.7)
Small-cap pharma/biotech ($250 million–$2 billion market capitalization)	17 (19.3)
Micro-cap pharma/biotech (≤$250 million market capitalization)	5 (5.7)
CMC vendor/CMO	15 (17.0)
Title/role	
Senior or mid-level management	41 (46.6)
Technical expert or specialist involved in strategic decision-making	24 (27.3)
Executive or senior leadership	23 (26.1)
Primary specialization	
CMC	23 (26.1)
R&D	21 (23.9)
Pipeline or portfolio strategy	16 (18.2)
NPP	15 (17.0)
Combination products	13 (14.8)
Number of years of experience working in primary specialization	
3–5	9 (10.2)
6–10	26 (29.5)
11–20	44 (50.0)
≥21	9 (10.2)
Main therapeutic area of expertise	
Oncology	46 (52.3)
Neurology	11 (12.5)
Endocrinology	11 (12.5)
Rheumatology	7 (8.0)
Dermatology	4 (4.5)
Gastroenterology	4 (4.5)
Nephrology	1 (1.1)
Other (analgesics, women’s health, Central Nervous System, and ophthalmology specified in survey free-text response)	4 (4.5)
Rare disease experience	
Rare disease (≤700 people per million)	69 (78.4)
Ultra-rare disease (≤20 people per million)	2 (2.3)
Not applicable	17 (19.3)
Involvement in decision-making about SC combination product development	
Identified as decision-maker	25 (28.4)
Influenced decision-making process	63 (71.6)
Number of high-concentration SC biologic products worked on throughout career	
5	46 (52.3)
6–10	21 (23.9)
11–20	14 (15.9)
21–30	3 (3.4)
31–100	3 (3.4)
>100	1 (1.1)
Familiarity with creating high-concentration liquid formulations for SC delivery (1 = Minimal familiarity, 5 = Very familiar)	
3	10 (11.4)
4	27 (30.7)
5—Very familiar	51 (58.0)
Understanding of challenges associated with development of high-concentration formulations for SC delivery (1 = Minimal understanding, 5 = Deep understanding)	
3	7 (8.0)
4	34 (38.6)
5—Deep understanding	47 (53.4)
Familiarity with development and outcomes of biologics requiring large volumes (≥3 mL) for SC injection (1 = No familiarity, 5 = Very familiar)	
3	15 (17.0)
4	32 (36.4)
5—Very familiar	41 (46.6)
Number of instances of *direct* involvement with projects where biologic was deprioritized or abandoned due to challenges in achieving small volumes (≤3 mL) for SC administration	
0	8 (9.1)
1–2	38 (43.2)
3–5	30 (34.1)
6–10	6 (6.8)
11+	6 (6.8)
Number of instances of *indirect* involvement with projects where biologic was deprioritized or abandoned due to challenges in achieving small volumes (≤3 mL) for SC administration	
0 or do not know	14 (15.9)
1–2	42 (47.7)
3–5	25 (28.4)
6–10	5 (5.7)
11+	2 (2.3)

Abbreviations: CMC, Chemistry Manufacturing and Controls; CMO, contract manufacturing organization; mL, milliliters; NPP, new product planning; R&D, research and development; SC, subcutaneous.

### 3.2. Survey results

#### 3.2.1. Perceptions of SC volume capacity

When asked about the maximum volume that could be administered without a PE, 64.8% selected ≤ 3 mL for SC bolus administration, while 33.0% selected ≤ 3 mL for SC infusion over several minutes (Figure 4). The primary basis reported for participants’ perceptions of SC bolus volume was familiarity with commercialized biologics (44.3%), followed by expert or colleague opinion (25.0%), peer-reviewed literature (19.3%) and company materials or company representative from manufacturer of a permeation enhancer (8.0%). Similar responses were observed for SC infusion over several minutes: 35.2% cited familiarity with commercialized biologics, 26.1% expert or colleague opinion, 20.5% peer-reviewed literature, and 14.8% company materials or a company representative from a PE manufacturer. Three participants (3.4%) for each question cited a lecture at a conference/seminar.

**Figure 4.**
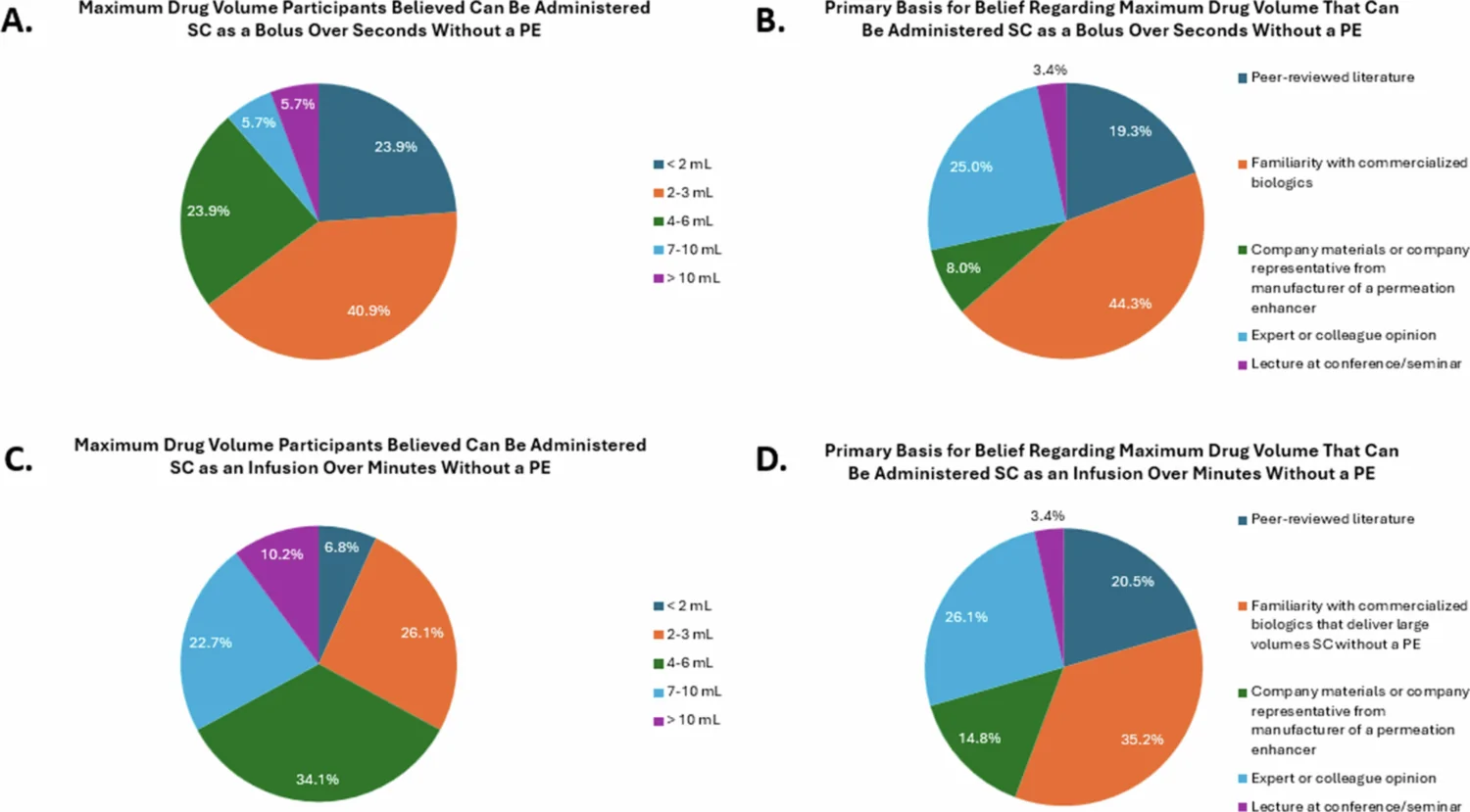
Respondents’ perceptions of maximum biologic volume that can be administered by SC bolus (A) or SC infusion over several minutes (C) without a PE and primary basis for these perceptions (B and D). Abbreviations: mL, milliliters; PE, permeation enhancer; SC, subcutaneous.

#### 3.2.2. Large-volume R&D asset development: reported experience

Most participants (90.9%, *n* = 80) reported direct involvement in at least one project in which a drug was deprioritized or abandoned due to challenges in achieving small (≤3 mL) volumes for SC administration. Among these respondents, the mean number of such projects was 4.08 (median 3; mode 2). Respondents estimated that a weighted mean of 32.4% of relevant drugs had been abandoned and 28.9% had been deprioritized because of challenges achieving small volumes for SC administration (Figure 5; Table 4). The primary reasons selected for abandoning a drug rather than pursuing a larger-volume SC approach were lack of experience with large-volume formulations or devices (29.5%), patient and/or HCP preference (19.3%), development cost or timeline (17.0%), patient comfort/convenience (14.8%), insufficient or uncertain market demand for large-volume formulations (12.5%), competitive landscape (2.3%), and treatment adherence (1.1%). Three participants (3.4%) reported other reasons, including formulation failures, insufficient bioavailability, and increased costs associated with studying large-volume administration. The majority of participants (63.6%) reported that large-volume drugs were only pursued if they offered distinct clinical benefits or addressed a high unmet need within the therapeutic area regardless of competition from small-volume or oral options. A further 31.8% reported that large-volume drugs are more likely to be pursued when competition from small-volume or oral options is low, and 4.5% reported that the level of competition does not significantly affect the decision.

**Figure 5.**
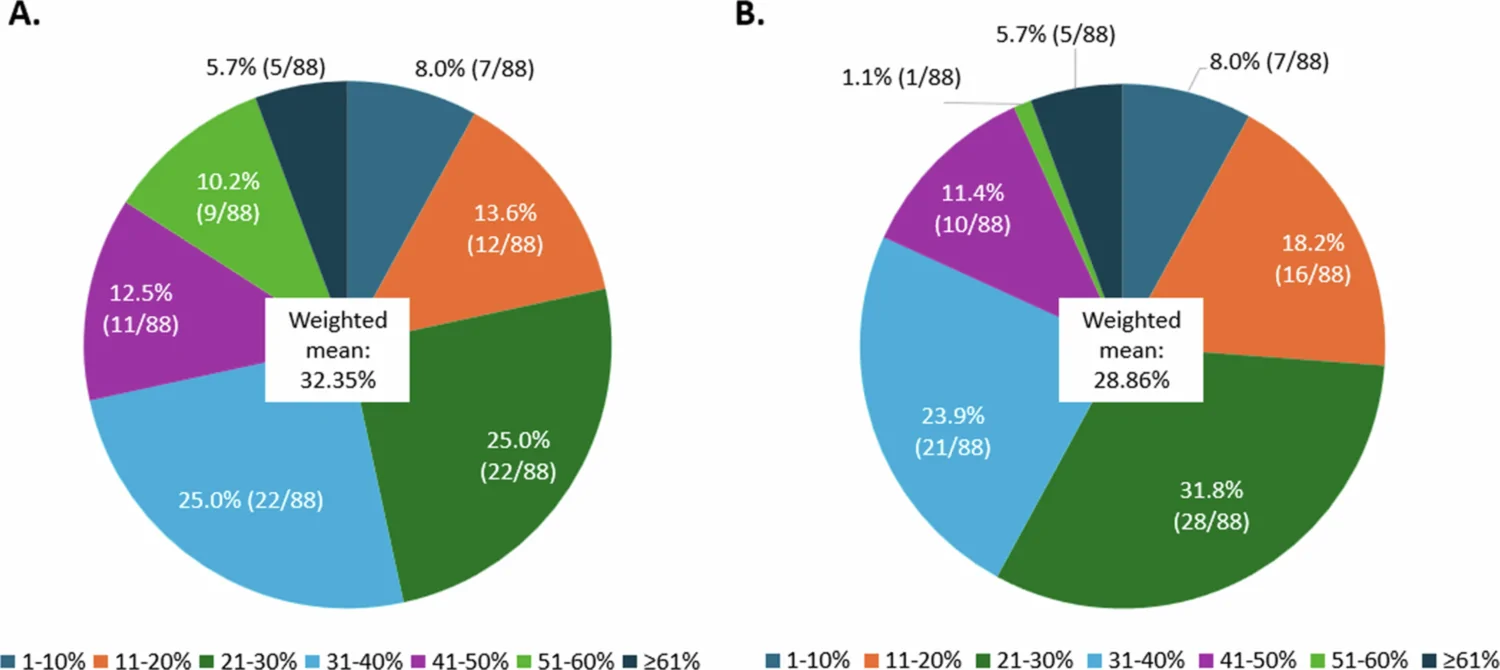
Respondent-estimated proportion of biologics abandoned (A) or deprioritized (B) due to challenges in achieving small (≤3 mL) subcutaneous administration volumes.

Respondents’ estimates of the proportion of deprioritized or abandoned drugs that might be well-suited for further development with a drug-delivery system without viscosity-related limitations varied widely, with a weighted mean of 36.6% (mode 31-40%) (Figure 6). The weighted means and modes for volume, viscosity, and concentration at which respondents reported that development became a concern for SC biologic drugs are shown in Table 3.

**Table 3.** Respondent-reported thresholds at which development becomes a concern for subcutaneous biologic drugs.

Characteristic (unit)	Weighted mean	Mode
Volume (mL)	5.67	4-6
Viscosity (cP)	16.56	19-22
Concentration (mg/mL)	147.01	101-125

Abbreviations: cP, centipoise; mg, milligrams; mL, milliliters.

**Figure 6.**
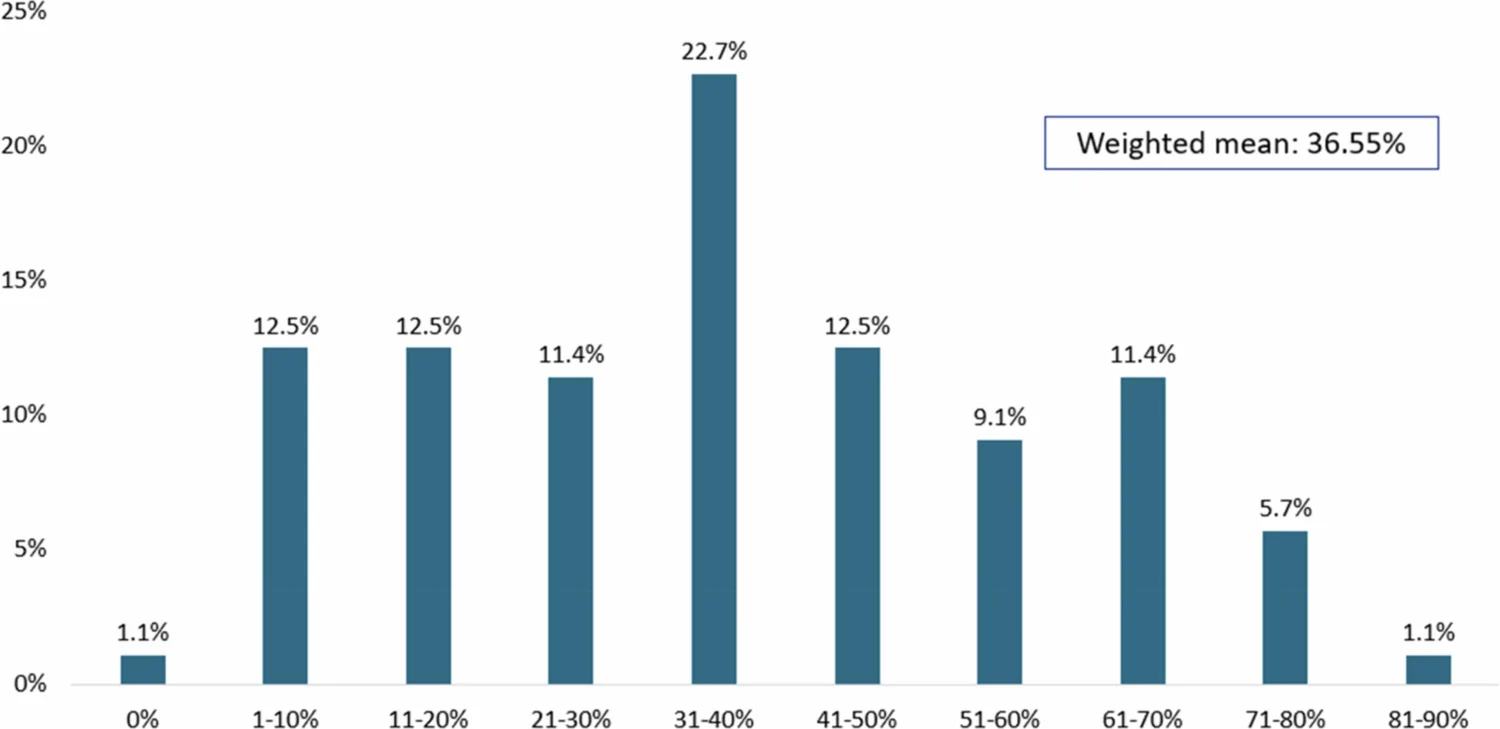
Respondent-estimated percentage of biologics deprioritized or abandoned that might be well-suited for further development with a drug delivery system without viscosity-related limitations.

#### 3.2.3. Potential development opportunities

Respondents whose primary specialization was early R&D or CMC (n = 44) estimated the proportion of biologics deprioritized or abandoned because of challenges achieving small SC volumes for which a longer dosing interval might have been possible with a larger-volume delivery approach; the weighted mean estimate was 25.0% (Table 4) (mode 11-20%, Figure 7). Participants rated the commercial potential of biologics that were deprioritized or abandoned due to challenges with large volumes for SC administration a mean of 2.98 (median 3, mode 3) on a scale of 1 (low potential) to 4 (high potential); no respondent rated it 1 (low). (Table 4).

**Table 4.** Participants rated the commercial potential of biologics that were deprioritized or abandoned due to challenges with large v olumes for SC administration a mean of 2.98 (median 3, mode 3) on a scale of 1 (low potential) to 4 (high potential); no respo ndent rated it 1 (low).

Outcome	Weighted mean (%)	Median (%)	Mode (%)
Respondent-estimated percentage of biologics abandoned due to challenges in achieving small volumes	32.4	31.3 (95% CI 27.3-35.4)	21-30/31-40
Respondent-estimated percentage of biologics deprioritized due to challenges in achieving small volumes	28.9	27.8 (95% CI 24.9-31.8)	21-30
Respondent-estimated percentage of biologics deprioritized or abandoned due to challenges with viscosity limits for SC administration that would be well-suited for further development with a drug delivery system without viscosity-related limitations	36.6	25.1 (95% CI 18.8-32.1)	31-40
Respondent-estimated percentage of biologics deprioritized or abandoned due to challenges in achieving small SC volumes that could have had their dosing intervals extended with a large-volume delivery device	25.0	26.8 (21.2-32.3)	11-20

**Figure 7.**
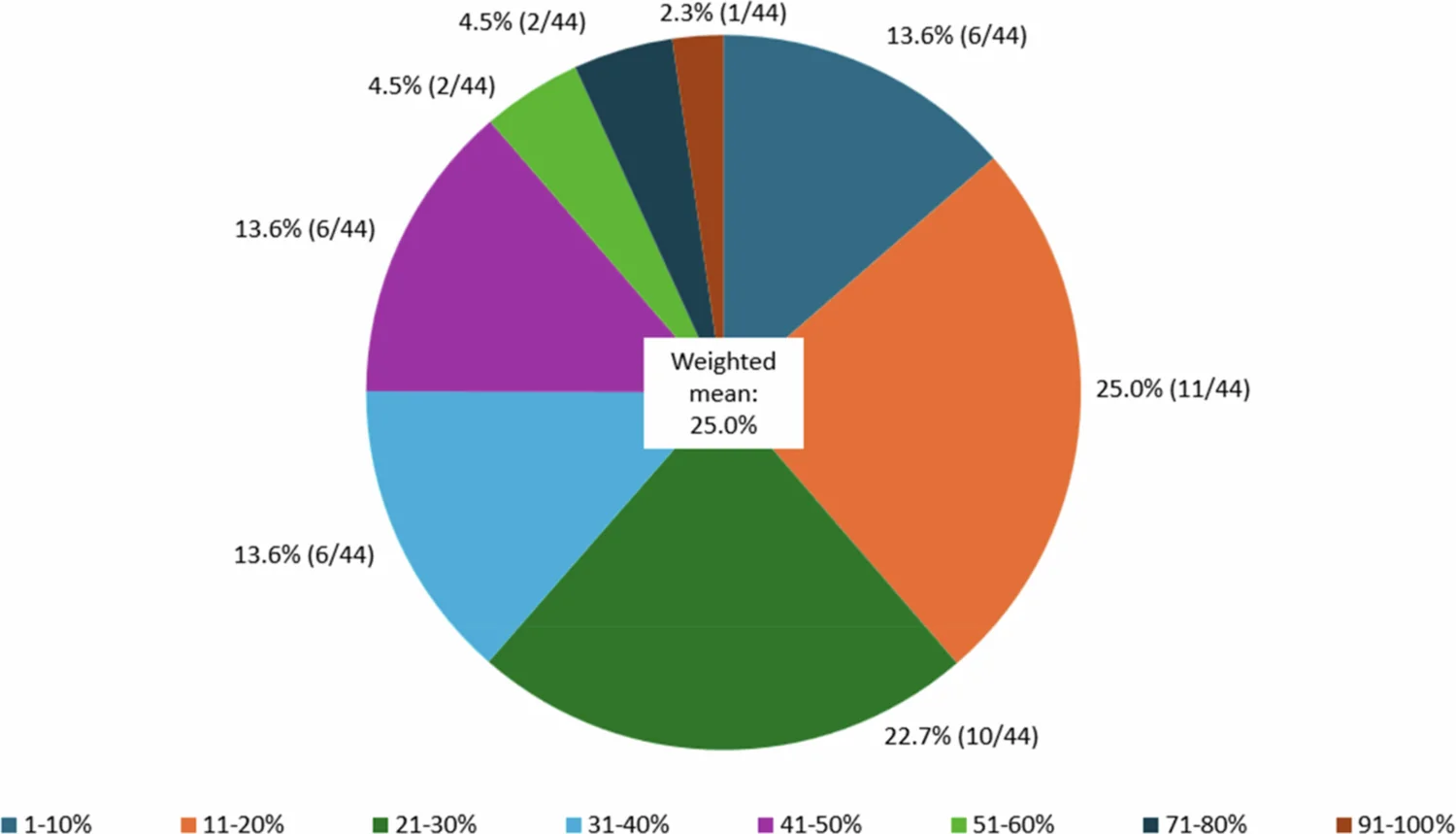
Respondent-estimated proportion of biologics deprioritized or abandoned due to challenges achieving small volumes for which an extended dosing interval might have been possible with a larger-volume delivery approach.

#### 3.2.4. Reconsideration of deprioritized or abandoned large-volume R&D assets

When asked whether their organization maintained an archive or database of deprioritized or abandoned biologics, 34.1% responded yes, 26.1% said no, 29.5% did not know, and 10.2% reported that the question was not applicable. The department reported to lead decision-making regarding reconsideration of deprioritized or abandoned biologics was most commonly R&D (30.7%), followed by senior leadership or an executive steering committee (22.7%), commercial/new product planning (18.2%), business development (15.9%), and CMC (12.5%). Participants rated their organizations’ openness to revisiting previously deprioritized or abandoned biologics a mean of 2.95 (median 3, mode 3) on a scale of 1 (not at all open) to 4 (extremely open); 15 participants responded that they were not sure.

## 4. Discussion

In this sample of 88 industry professionals with substantial experience in SC biologic development, perceptions of feasible SC administration volume varied. Nearly all respondents (*n* = 80, 90.9%) reported direct involvement in at least one project in which difficulty achieving a “small” (≤3 mL) SC volume had contributed to deprioritization or abandonment. This proportion partly reflects the study eligibility criteria, which required participants to report either direct or indirect experience with projects affected by SC volume constraints, and should not be interpreted as an estimate of the prevalence of such experience among industry professionals more broadly. However, indirect involvement alone was sufficient for inclusion, and only 8 respondents (9.1%) qualified on that basis. Within this sample, most respondents had therefore encountered volume-related deprioritization or abandonment firsthand rather than only secondhand. Respondents also estimated, from their own past projects, how often volume-related challenges had led to a biologic being deprioritized or abandoned. This study represents the first systematic investigation of decision-making processes regarding large-volume SC drug candidates among pharmaceutical and biotechnology industry professionals involved in early-stage drug development.

Approximately one-third of participants selected ≤ 3 mL as the maximum volume for SC infusion over several minutes without a PE. This contrasts with the availability of SC products administered at substantially greater volumes without a PE and suggests that a ≤ 3 mL threshold may be generalized beyond the administration contexts in which it is most relevant. This does not imply that an approximately 2-3 mL practical constraint is irrelevant to all SC delivery. The amount of drug product that can be delivered SC is determined by a combination of anatomical, device, formulation, and administration factors, while systemic exposure is determined by the dose administered and the proportion that reaches systemic circulation.

This distinction is also relevant when comparing the present findings with previous surveys in which substantial proportions of formulation professionals selected ≤ 3 mL as the maximum volume for SC bolus administration or infusion over several minutes (Desai et al. 2025a, Desai et al. 2025b, 2026). Together, these findings highlight variability in how feasible SC administration volume is interpreted across industry and support a more context-specific assessment of volume during development rather than reliance on a single threshold.

Respondents most commonly identified limited experience with large-volume formulations or devices, perceived patient and/or HCP preference, and development cost or timeline as reasons for not pursuing larger-volume SC approaches. Although user-related considerations (i.e. patient and/or HCP preference, patient comfort/convenience, and treatment adherence) were collectively selected more often than any single reason, limited experience was the most frequently selected individual reason. This suggests that respondents’ perceptions of feasible administration volumes are influenced, at least in part, by limited familiarity with larger-volume biologics rather than by a universal upper limit. This finding further supports evaluating feasible volume in the context of the specific product, formulation, device, administration approach, and patient population rather than assuming a fixed volume threshold. Respondents’ rationales for their estimates of maximum feasible bolus or infusion volume support this interpretation: estimates were most often reported to be based on experience with marketed biologics or on expert or colleague opinion rather than on peer-reviewed literature. Perceived volume limits may therefore be shaped largely by precedent from a limited number of existing products. Because those products reflect earlier development choices and prevailing volume conventions, using them as benchmarks may reinforce volume-related assumptions even when direct physiological evidence for a fixed limit is lacking.

Most respondents reported that large-volume biologics were pursued when they offered distinct clinical benefits or addressed high unmet needs despite competition from small-volume or oral options. Most of the remainder indicated that large-volume candidates are more likely to be pursued when such competition is low, and very few considered competition to be an insignificant factor. Taken together, these responses suggest that clinical differentiation functions as a prerequisite for advancing large-volume SC candidates and that administration volume operates as a screening criterion in asset-level decisions rather than as a neutral design variable.

Respondentsin early R&D and CMC estimated that nearly 30% of biologics deprioritized or abandoned because of challenges achieving small SC volumes might have permitted a longer dosing interval if a larger administration volume were feasible. This suggests that administration volume and dosing frequency may warrant consideration together during development rather than as independent constraints. However, the survey did not evaluate individual products or compare the clinical performance of specific delivery technologies, and no conclusions regarding the superiority of a particular device or delivery approach can therefore be drawn from these data.

When asked to set aside volume-related challenges, most respondents rated the commercial potential of assets deprioritized or abandoned for volume reasons as moderate to high, and none rated it low. A majority also described their organizations as open or very open to revisiting such programs, although a meaningful minority did not and some were unsure. These observations suggest that changes in formulation capabilities, delivery options, competitive dynamics, or development priorities may create opportunities to reassess selected programs over time.

Formulation- and device-based strategies represent complementary approaches to addressing SC administration volume constraints. The present survey was not designed to compare the clinical effectiveness, development burden, or suitability of PE coformulation with specific large-volume delivery technologies. Selection among these approaches should therefore be based on the characteristics and requirements of the individual product, including formulation, pharmacokinetics, dose, administration conditions, and intended user population.

Respondents to this survey reported that almost a third of potential biologics are abandoned or deprioritized due to challenges in achieving small SC volumes, but participants in this study considered the commercial potential of these biologics as well as the openness of their organizations to revisiting and potentially resurrecting such biologics to be high. It is possible that, due to the prevalence of misunderstanding regarding SC volume limits, promising new therapies are abandoned or deprioritized unnecessarily. Furthermore, the most common reasons cited for abandoning or deprioritizing biologics due to challenges in achieving small volume were a lack of experience with large-volume formulations or devices and patient and/or HCP preference. These knowledge gaps may result in reduced R&D productivity within biopharmaceutical organizations and, more critically, limit patient access to potentially transformative therapies. Interestingly, most participants reported that large-volume biologics are only pursued if they offer distinct clinical benefits or address a high unmet need within the therapeutic area regardless of competition from small-volume or oral options. This suggests that large-volume SC biologics may be held to a higher standard than small-volume SC biologics. Biologic candidates that are incompatible with PEs such as hyaluronidase may be deprioritized or abandoned during SC development, and efforts to evaluate or resolve PE compatibility can introduce additional development delays (Desai et al. 2026).

Several companies are already demonstrating how overcoming volume limitation with improved dosing and delivery rather than efficacy or safety can result in competitive differentiation by increasing dosing intervals. For example, Serina therapeutics is competing with a twice-weekly SC administration with their POZ technology using an OBI for delivery (BioSpace 2025) against a 24/7 pump from Abbvie (Buntz 2025). Similarly, Alexion’s IV ravulizumab requires less frequent infusion at 8-week intervals (Alexion Pharmaceuticals 2018) than its IV eculizumab (also used to treat paroxysmal nocturnal hemoglobinuria), (European Medicines Agency (EMA) 2023) which must be administered weekly and then every two to three weeks, amounting to 19.5 fewer infusions annually per patient. Other examples of this approach include Apogee Therapeutics’ interleukin (IL) 13 (IL-13) antibody, (Waldron 2025) Spyre Therapeutics’ tumor necrosis factor-like cytokine 1 A (TL1A) and IL-23 antibodies, (Spyre Therapeutics 2025) Viridian Therapeutics’ insulin-like growth factor receptor 1 (IGF-1R) antibody, (Foster et al. 2024) and Oruka Therapeutics’ IL-23p19 and IL-17A/F antibodies (BioSpace 2024). These examples demonstrate that extending dosing intervals can require larger SC doses and therefore larger volumes.

The formulation and delivery barriers identified in this survey are particularly consequential for antibody-drug conjugates (ADCs), a rapidly growing drug class where every Food and Drug Administration (FDA)-approved product is administered IV. The class now includes more than 15 FDA-approved products with over 400 in clinical development (Chen et al. 2025). All but one approved ADC (mirvetuximab soravtansine) are formulated as lyophilized powders requiring reconstitution (Wen et al. 2025). The IV-to-SC conversion opportunity for ADCs is significant, as preclinical data have demonstrated feasibility of SC ADC delivery both with and without the use of a PE (Wen et al. 2025). Because raising concentration is particularly difficult for this class, ADCs are an especially important drug class in which treating volume as a fixed threshold could hinder SC development. Given that over 90% of respondents in the current survey reported direct involvement in projects where drugs were deprioritized or abandoned due to volume constraints, the implications for an entirely IV-dependent drug class with over 400 candidates in clinical development are substantial.

This study has several limitations. First, the eligibility criteria intentionally restricted participation to professionals with self-reported experience in high-concentration and large-volume SC biologic development and related decision-making, thereby ensuring that responses were informed by relevant expertise. However, participation among eligible professionals was voluntary, and respondents may have differed systematically from eligible individuals who did not participate. Accordingly, the findings should be interpreted as reflecting the perspectives of this experienced respondent group rather than as representative of the wider biopharmaceutical industry or necessarily of all professionals with relevant expertise. Second, the findings are based on retrospective self-report and are susceptible to selection, recall, and response bias. Estimates of the proportions of biologics abandoned or deprioritized were respondents’ perceptions and were not verified against independently identified biologic development records; individual respondents may also have had experience with overlapping development programs. Third, the cross-sectional study design cannot establish that perceptions of administration volume caused individual development decisions. Fourth, the investigator-developed questionnaire did not undergo cognitive testing or formal validation before administration. Finally, participant responses regarding patient and HCP preference reflect developers' perceptions rather than preferences measured directly. Administration preferences are context-dependent, varying with disease, treatment setting, dosing frequency, and available alternatives. Because patients and HCPs were not surveyed, the present data cannot establish whether an assumed preference for smaller volumes is held by end users or how it is weighed against attributes such as injection frequency or time in clinic. Studies incorporating direct patient and HCP input are needed to clarify how administration volume, duration, frequency, and delivery modality contribute to treatment acceptability and, in turn, to development decisions.

Strengths of the study include the substantial relevant experience of the respondents, representation across multiple industry functions and geographical regions, and detailed responses concerning both technical and organizational considerations in SC biologic development. The study also provides insight into questions that have received limited attention in the published literature, particularly in an area where commercially sensitive information and corporate confidentiality agreements may restrict public disclosure of development experience and decision-making.

## 5. Conclusions

Among this selected sample of industry professionals with substantial experience in SC biologic drug development, perceptions of feasible SC administration volume varied, and respondents reported that challenges achieving small SC volumes had led to abandonment or deprioritization of promising assets. Approximately one-third selected ≤ 3 mL as the maximum volume for SC infusion over several minutes without a PE despite the availability of products administered at substantially greater volumes. Moreover, most respondents reported that large-volume biologics are only pursued if they offer distinct clinical benefits or address high unmet needs, regardless of competition from small-volume or oral alternatives. These findings highlight the importance of distinguishing the capacity limits of a particular device or administration method from the biological limits of SC tissue, which can accommodate considerably larger volumes. More nuanced, product-specific evaluation of these factors may broaden the range of feasible SC development strategies while ensuring that administration decisions remain grounded in clinical and user needs. Prospective or biologic-level studies are needed to quantify the contribution of administration volume considerations to development decisions and evaluate the outcomes of alternative strategies.

## Supplementary Material

Supplementary Material Supplementary Information

## Data Availability

The data collected for the purposes of this manuscript are available from the corresponding author upon reasonable request.

## Previous presentation

CRS July 2025; AAPS NBC May 2025; AACR April 2025.
