# Supplementary material for: The impact of perceived volume limits on subcutaneous biologic drug development: an industry survey
Source: Drug Deliv. 2026 Nov 7;33(1):2756024. doi: 10.1080/10717544.2026.2756024 (PMC13757793; doi:10.1080/10717544.2026.2756024)
Supplement: Supplementary file 1 [file IDRD_A_2756024_SM5997.docx]

**Supplementary Information**

**[SCREENING]**

1. In which country do you currently work/reside? [Country Dropdown]
2. Which of the following best describes your current organization?
   a. Large-cap Biotech/Pharma (>$10 billion market capitalization)
   b. Mid-cap Biotech/Pharma ($2 – $10 billion market capitalization)
   c. Small-cap Biotech/Pharma ($250 million – $2 billion market capitalization)
   d. Micro-cap Biotech/Pharma (<250 million)
   e. Contract Development & Manufacturing Organization (e.g., Coriolis Pharma, Lonza Group, Catalent, Charles River Laboratories, Wuxi, and others)
   f. Contract Research Organization
   g. Independent Chemistry, Manufacturing, & Controls Consultant
   h. Other, please specify: [Free Text]
3. What best describes your primary area of specialization?
   a. New Product Planning (NPP)
   b. Early Research & Development (R&D)
   c. Pipeline or Portfolio Strategy
   d. Medical Affairs [Terminate]
   e. Commercial [Terminate]
   f. Chemistry, Manufacturing and Controls (CMC)
   g. Combination Product
   h. Other, please specify [Free Text]
4. How many years of experience do you have working in the field of [PIPE S3] [≤2 = Terminate]?
5. Which of the following best describes your current role within the organization?
   a. Executive or Senior Leadership (e.g., Vice President, Executive Director)
   b. Senior or Mid-Level Management (e.g., Senior Director, Director, or equivalent)
   c. Technical Expert or Specialist involved in strategic decision-making (e.g., Associate Director, Senior Manager, Manager)
   d. Other, please specify [Free Text]
6. On a scale of 1-5 with 1 being minimal familiarity to 5 being very familiar, please rate your familiarity with the development of high-concentration formulations (≥100 mg/mL) for subcutaneous delivery [≤ 2 = Terminate]
7. On a scale of 1-5 with 1 being minimal understanding to 5 being deep understanding, please rate your understanding of the challenges associated with the development of high-concentration formulations (≥100 mg/mL) for subcutaneous delivery [≤ 2 = Terminate]
8. How many subcutaneous, high-concentration (≥100 mg/mL) drug products have you worked on during your career? [≤4 = Terminate]
9. On a scale of 1 to 5, with 1 being not familiar to 5 being very familiar, how familiar are you with the development and outcomes of drugs requiring large volumes for subcutaneous injection? (≥ 3 mL) [≤ 2 = Terminate]
10. Have you been directly involved in projects where a drug was deprioritized or abandoned due to challenges in achieving small volumes (≤ 3 mL) for subcutaneous administration?
    a. Yes [If chosen, ‘How many instances?’ free text box]
    b. No
    c. I don’t know
11. Have you been indirectly involved in projects where a drug was deprioritized or abandoned due to challenges in achieving small volumes (≤ 3 mL), for subcutaneous administration?
    a. Yes, how many instances? [open text] [If ‘B’ on S10 = Limit to 12 per each specialization group (e.g., Early R&D, CMC, NPP/Portfolio Strategy, Combo Product]
    b. No [If ‘B’ or ‘C’ on S10 = Terminate]
    c. I don’t know [If ‘B’ or ‘C’ on S10 = Terminate]
12. What is the main therapeutic area of your experience with subcutaneous drug products?
    a. Oncology
    b. Neurology
    c. Nephrology
    d. Dermatology
    e. Gastroenterology
    f. Rheumatology
    g. Endocrinology
    h. Benign Hematology
    i. Primary Care (e.g. Obesity)
    j. Other, please specify [Free Text]
13. Please indicate if your main therapeutic area of experience falls within one of the categories below:
    a. Rare Disease (defined as affecting 700 people per million or fewer)
    b. Ultra-rare Disease (defined as affecting 20 people per million or fewer)
    c. Not Applicable
14. How extensively have you contributed to decision-making processes regarding the development of subcutaneous drug products, either presently or in the past?
    a. I am/was the decision-maker
    b. I influence(d) the decision-making process
    c. I am/was not involved in the decision-making process [Terminate]
    d. I am not sure [Terminate]

**[MAIN SURVEY]**

1. What is the maximum drug volume that you believe can be administered subcutaneously, as an immediate bolus (all-at-once), without hyaluronidase (e.g., rHuPH20) as a permeation enhancer?
   a. < 2 mL
   b. 2 – 3 mL
   c. 4 – 6 mL
   d. 7 – 10 mL
   e. > 10 mL
2. What is the primary basis for your above answer?
   a. Peer-reviewed literature
   b. Familiarity with commercialized biologics
   c. Company materials or company representative from manufacturer of a permeation enhancer
   d. Expert or colleague opinion
   e. Lecture at conference/seminar
   f. Other, please specify: [Open Text]
3. What is the maximum drug volume that you believe can be administered subcutaneously, as a subcutaneous infusion (over several minutes), without hyaluronidase (e.g., rHuPH20) as a permeation enhancer?
   a. < 2 mL
   b. 2 – 3 mL
   c. 4 – 6 mL
   d. 7 – 10 mL
   e. > 10 mL
4. What is the primary basis for your above answer?
   a. Peer-reviewed literature
   b. Familiarity with commercialized biologics that deliver large volumes subcutaneously without a permeation enhancer
   c. Company materials or company representative from manufacturer of a permeation enhancer
   d. Expert or colleague opinion
   e. Lecture at conference/seminar
   f. Other, please specify: [Open Text]
5. What percentage of potential drugs are ABANDONED due to challenges in achieving small volumes (≤ 3 mL) for subcutaneous administration?
   a. 0%
   b. 1-10%
   c. 11-20%
   d. 21-30%
   e. 31-40%
   f. 41-50%
   g. 51-60%
   h. 61-70%
   i. 71-80%
   j. 81-90%
   k. 91-100%
   l. Other [If chosen, ‘please specify’ free text box]
6. What is the primary reason organizations might choose to **ABANDON** a potential drug that struggles with achieving small-volumes (≤ 3 mL) for subcutaneous administration, versus exploring options for large-volume administration?
   a. Insufficient or uncertain market demand for large-volume formulations
   b. Patient and/or healthcare provider preference
   c. Lack of experience with large-volume formulations or devices
   d. Patient comfort/convenience
   e. Development cost or timeline
   f. Competitive landscape
   g. Treatment adherence
   h. Other, please specify [Free Text]
7. What percentage of potential drugs are DEPRIORITIZED due to challenges in achieving small volumes (≤ 3 mL) for subcutaneous administration?
   a. 0%
   b. 1-10%
   c. 11-20%
   d. 21-30%
   e. 31-40%
   f. 41-50%
   g. 51-60%
   h. 61-70%
   i. 71-80%
   j. 81-90%
   k. 91-100%
   l. Other [If chosen, ‘please specify’ free text box]
8. How does the level of competition, such as availability of small-volume and/or oral options, within a therapeutic area impact the decision to advance a large-volume drug?
   a. Large-volume drugs are more likely to be pursued when there’s low competition versus small-volume and/or oral options
   b. Large-volume drugs are only pursued if they offer distinct clinical benefits or address a high unmet need within the therapeutic area, regardless of competition from small-volume or oral options
   c. The level of competition versus small-volume and/or oral options does not significantly impact the decision to pursue a large-volume drug
   d. Other, please specify [Free Text]
9. [If ‘B’ or ‘F’ on S3] What percentage of potential drugs, deprioritized OR abandoned due to challenges in achieving small volumes (≤ 3 mL) for subcutaneous administration, could have had their dosing intervals extended with a large-volume delivery device, such as an on-body delivery system that can deliver up to 25 mL?
   a. 0%
   b. 1-10%
   c. 11-20%
   d. 21-30%
   e. 31-40%
   f. 41-50%
   g. 51-60%
   h. 61-70%
   i. 71-80%
   j. 81-90%
   k. 91-100%
   l. Other [If chosen, ‘please specify’ free text box]
10. What percentage of potential drugs are deprioritized OR abandoned due to challenges in achieving a certain viscosity for subcutaneous administration?
    a. 0%
    b. 1-10%
    c. 11-20%
    d. 21-30%
    e. 31-40%
    f. 41-50%
    g. 51-60%
    h. 61-70%
    i. 71-80%
    j. 81-90%
    k. 91-100%
    l. Other [If chosen, ‘please specify’ free text box]
11. What percentage of potential drugs, deprioritized OR abandoned due to challenges with viscosity limits for subcutaneous administration, would be well-suited for further development with a drug delivery system that doesn’t have viscosity-related limitations?
    a. 0%
    b. 1-10%
    c. 11-20%
    d. 21-30%
    e. 31-40%
    f. 41-50%
    g. 51-60%
    h. 61-70%
    i. 71-80%
    j. 81-90%
    k. 91-100%
    l. Other [If chosen, ‘please specify’ free text box]
12. On a scale of 1 to 4, with 1 being low potential and 4 being high potential, how would you rate the commercial potential of drugs that were deprioritized or abandoned solely due to challenges with large drug volumes for subcutaneous administration? In other words, how promising were these molecules when you don’t consider their volume-related challenges?
13. At what VOLUME limit does a potential drug for subcutaneous administration become a concern for its future development?
    a. < 2 mL
    b. 2 – 3 mL
    c. 4 – 6 mL
    d. 7 – 10 mL
    e. > 10 mL
    f. Other, please specify [Free Text]
14. [IF S3 = b, f, OR g] At what VISCOSITY limit does a potential drug for subcutaneous administration become a concern for its future development?
    a. < 3 cP
    b. 3 – 6 cP
    c. 7 – 10 cP
    d. 11 – 14 cP
    e. 15 – 18 cP
    f. 19 – 22 cP
    g. 23 – 26 cP
    h. 27 – 30 cP
    i. > 30 cP
    j. Other, please specify [Free Text]
15. [IF S3 = b, f, OR g] At what CONCENTRATION limit does a potential drug for subcutaneous administration become a concern for its future development?
    a. 0 – 25 mg/mL
    b. 26 – 50 mg/mL
    c. 51 – 75 mg/mL
    d. 76 – 100 mg/mL
    e. 101 – 125 mg/mL
    f. 126 – 150 mg/mL
    g. 151 – 175 mg/mL
    h. 176 – 200 mg/mL
    i. 201 – 225 mg/mL
    j. 226 – 250 mg/mL
    k. 251 – 275 mg/mL
    l. 276 – 300 mg/mL
    m. > 300 mg/mL
    n. Other, please specify [Free Text]
16. Does your organization maintain an archive or database for drugs that have been deprioritized or abandoned during development?
    a. Yes [If chosen, ‘Please provide details on how this information is organized and accessed’ free text box]
    b. No
    c. I don’t know
    d. Not applicable
17. [If ‘A’ on Screening Question 10] Which functional team is responsible for maintaining and managing access to an archive or database of drugs that have been deprioritized or abandoned during development? [Free Text]
18. Who typically leads the decision-making process for reviving a drug that has been deprioritized or abandoned?
    a. Research & Development (R&D)
    b. Commercial & New Product Planning (NPP)
    c. Chemistry, Manufacturing, and Controls (CMC)
    d. Business Development (BD)
    e. Senior Leaders or Executive Steering Committee
    f. Other, please specify [Free Text]
19. In your firsthand experience, during the development of a subcutaneous drug, what percentage of your colleagues typically support larger volume SC delivery (> 3 mL)?
    a. 0%
    b. 1-10%
    c. 11-20%
    d. 21-30%
    e. 31-40%
    f. 41-50%
    g. 51-60%
    h. 61-70%
    i. 71-80%
    j. 81-90%
    k. 91-100%
    l. Other [If chosen, ‘please specify’ free text box]
20. On a scale of 1 to 4, with 1 being not open at all and 4 being extremely open [provide an option for “not sure”], how willing is your organization to revisit and potentially resurrect drugs that have previously been deprioritized or abandoned? [If ≤2 chosen, ‘What factors would need to change for this to happen more frequently?’ free text box]
21. How familiar are you with the following subcutaneous injection devices and technologies? *Please rate your familiarity on a scale of 1 to 5, where 1 is not at all familiar and 5 is extremely familiar*

|  | **1- Not at all Familiar** | **2 - Slightly Familiar** | **3 - Somewhat Familiar** | **4 - Moderately Familiar** | **5 - Extremely Familiar** |
| --- | --- | --- | --- | --- | --- |
| SC Syringe Pump | 🔾 | 🔾 | 🔾 | 🔾 | 🔾 |
| On-Body Delivery System | 🔾 | 🔾 | 🔾 | 🔾 | 🔾 |
| Permeation Enhancers (e.g., hyaluronidase) | 🔾 | 🔾 | 🔾 | 🔾 | 🔾 |
